# Parthenogenetic *vs*. sexual reproduction in oribatid mite communities

**DOI:** 10.1002/ece3.5303

**Published:** 2019-05-29

**Authors:** Mark Maraun, Tancredi Caruso, Jonathan Hense, Ricarda Lehmitz, Levan Mumladze, Maka Murvanidze, Ioana Nae, Julia Schulz, Anna Seniczak, Stefan Scheu

**Affiliations:** ^1^ JFB Institute of Zoology and Anthropology Georg August University Göttingen Göttingen Germany; ^2^ School of Biological Sciences and Institute for Global Food Security Queen's University of Belfast Belfast Northern Ireland; ^3^ Fachdidaktik Biologie, Nees‐Institut Rheinische Friedrich‐Wilhelms Universität Bonn Bonn Germany; ^4^ Senckenberg Museum of Natural History Görlitz Görlitz Germany; ^5^ Institute of Ecology and Institute of Zoology Ilia State University Tbilisi Georgia; ^6^ Institute of Entomology of Agricultural University of Georgia Tbilisi Georgia; ^7^ Emil Racovita Institute of Speleology of Romanian Academy Bucharest Romania; ^8^ Realfagbygget University Museum of Bergen, University of Bergen Bergen Norway

**Keywords:** oribatid mites, Red Queen, sexual reproduction, Structured Resource Theory of Sex, Tangled Bank, thelytoky

## Abstract

The dominance of sex in Metazoa is enigmatic. Sexual species allocate resources to the production of males, while potentially facing negative effects such as the loss of well‐adapted genotypes due to recombination, and exposure to diseases and predators during mating. Two major hypotheses have been put forward to explain the advantages of parthenogenetic versus sexual reproduction in animals, that is, the Red Queen hypothesis and the Tangled Bank/Structured Resource Theory of Sex. The Red Queen hypothesis assumes that antagonistic predator—prey/ parasite–host interactions favor sex. The Structured Resource Theory of Sex predicts sexual reproduction to be favored if resources are in short supply and aggregated in space. In soil, a remarkable number of invertebrates reproduce by parthenogenesis, and this pattern is most pronounced in oribatid mites (Oribatida, Acari). Oribatid mites are abundant in virtually any soil across very different habitats, and include many sexual and parthenogenetic (thelytokous) species. Thereby, they represent an ideal model group to investigate the role of sexual versus parthenogenetic reproduction across different ecosystems and habitats. Here, we compiled data on oribatid mite communities from different ecosystems and habitats across biomes, including tropical rainforests, temperate forests, grasslands, arable fields, salt marshes, bogs, caves, and deadwood. Based on the compiled dataset, we analyzed if the percentage of parthenogenetic species and the percentage of individuals of parthenogenetic species are related to total oribatid mite density, species number, and other potential driving factors of the reproductive mode including altitude and latitude. We then interpret the results in support of either the Red Queen hypothesis or the Structured Resource Theory of Sex. Overall, the data showed that low density of oribatid mites due to harsh environmental conditions is associated with high frequency of parthenogenesis supporting predictions of the Structured Resource Theory of Sex rather than the Red Queen hypothesis.

## 1 INTRODUCTION

The dominance of sexual reproduction in most animal taxa remains an enigma (Lehtonen, Jennions, & Kokko, 2012; Meirmans, Meirmans, & Kirkendall, 2012; Neiman, Lively, & Meirmans, 2017). Parthenogenetic reproduction has advantages that should, in theory, result in rapid replacement of sexual taxa. The advantages include doubled reproductive potential, no exposure to hazards during mating, easier colonization of new habitats, and maintenance of favorable gene combinations (Maynard Smith, 1978; Mirzaghaderi & Hörandl, 2016). A number of theories have been put forward to explain the “paradox of sex,” but no consensus has been reached and some researchers doubt the existence of a unifying theory (Neiman et al., 2017; West, Lively, & Read, 1999). Among theories, those proposing short‐term generational advantages of sexual reproduction have received most attention (Lehtonen et al., 2012), while theories based on long‐term multigenerational advantages and group selection arguments have lost appeal (Becks & Agrawal, 2011; Bell, 1982; West et al., 1999).

Two major theories based on short‐term advantages of sexual reproduction include (a) the Red Queen Theory (RQT), and (b) the Tangled Bank Theory (TBT) and Structured Resource Theory of Sex (SRTS), the latter two being based on similar mechanisms and therefore treated together here (Neimann et al., 2017; Song, Drossel, & Scheu, 2011). The RQT suggests that the genetic diversity generated by sexual reproduction reduces the ability of antagonists (parasites, predators) of species to adapt to host genotypes. It therefore predicts sexual taxa to dominate in habitats where parasite–host and/ or predator–prey interactions control community dynamics (Hamilton, 1980; Jaenike, 1978), which is often the case when predators/ parasites reach high densities (Ladle, 1992). The RQT does not make explicit predictions on the effects of resource availability, but it has been suggested that small populations are less infested by parasites (Arneberg, Skorping, Grenfell, & Read, 1998) thereby weakening Red Queen processes. A number of field and laboratory studies supported the RQT (Gibson & Fuentes, 2015; Haafke, Chakra, & Becks, 2016; Kotusz et al., 2014), but many are based on a single parasite–host system, the snail *Potamogyrgus antipodarum* and its trematode parasite (Jokela, Dybdahl, & Lively, 2009; King, Delph, Jokela, & Lively, 2009; Lively, 2010).

The TBT (Bell, 1982; Ghiselin, 1974) predicts sexual reproduction to dominate in spatially heterogeneous habitats where diverse niches are more likely to be successfully colonized by genetically diverse offspring produced via sexual reproduction. The TBT does not make explicit predictions on the role of biotic interactions or resource levels of the habitat of sexual versus parthenogenetic species. Building on the TBT, the SRTS explicitly includes resource dynamics and density‐dependent population regulation, but its predictions are essentially similar to those of the TBT (Scheu & Drossel, 2007). The SRTS predicts that sexual reproduction should become more frequent in spatially structured habitats where resources are structured and renewed at low rates. The implication of this theory is that density‐dependent population regulation is controlling the frequency of sexual and parthenogenetic reproduction. Both SRTS and RQT predict sexual reproduction to dominate when biotic interactions are strong, but the SRTS is based on bottom‐up control of population regulation due to resource limitation, whereas the RQT is based on top‐down control of prey species by predators and parasites. The SRTS explicitly predicts parthenogenesis to dominate in populations that are structured by harsh abiotic conditions since the advantage of sexual reproduction vanishes in the absence of resource limitation. The RQT makes no explicit predictions about the effects of environmental harshness, but harsh conditions typically reduce predator–prey/ parasite–host interactions thereby weakening the advantages of sexual reproduction. Importantly, the RQT and the SRTS are not mutually exclusive. In fact, their predictions partly overlap and some field observations might be explained by either of them (Neimann et al., 2017). Further, from the perspective of resources, parasites may be viewed as agents reducing the resource supply of the host; as a consequence, Red Queen processes may be viewed as forming part of the SRTS (Scheu & Drossel, 2007; Song et al., 2011).

The predictions of the two theories can be examined by studying the relative frequency of sexual or parthenogenetic reproduction in animal taxa across biomes and environmental gradients. These two theories can be tested in a number of soil animals including oribatid mites, collembolans, nematodes, earthworms, and isopods (Bell, 1982; Song et al., 2011). Arguably, the most powerful model taxon in this respect is oribatid mites, an abundant and diverse group of predominantly detritivorous and fungivorous soil arachnids. They occur in virtually any ecosystem including forests, grassland, tundra, taiga, agricultural sites, fresh water, bogs and marshes, and marine shorelines, and in a very wide range of microhabitats including different soil layers, decaying wood, fungal sporophores, epilithic, and epiphytic plants, bark of trees and suspended soils (Norton & Behan‐Pelletier, 2009).

Oribatid mites either reproduce sexually or via thelytoky; other reproductive modes such as arrhenotoky, cyclical parthenogenesis as well as geographic parthenogenesis are unknown (Norton, Kethley, Johnston, & O'Connor, 1993). About 10% of the known species of oribatid mites are assumed to be obligate parthenogenetic, with more than 90% of the individuals of oribatid mite communities in some ecosystems comprising parthenogenetic species (Maraun, Fronczek, Marian, Sandmann, & Scheu, 2013; Norton & Palmer, 1991). Norton and Palmer (1991) highlighted that parthenogenetic species dominate many early‐derivative taxa and, rather than each being recent derivatives of an ancestral sexual lineage, most extant parthenogenetic species form part of clusters of parthenogenetic species. These taxa represent phylogenetic groups of different size, from small to speciose, indicating past parthenogenetic radiations (Heethoff, Norton, Scheu, & Maraun, 2009; Pachl et al., 2017; Schaefer, Norton, Scheu, & Maraun, 2010). Furthermore, parthenogenetic species occur in habitats “where they should not be” according to most theories, for example, in climax habitats such as old‐growth forests (Huhta et al., 1986) and at low altitudes (Maraun et al., 2013). Finally, Cianciolo and Norton (2006) found a positive correlation between biotic interactions (competition and predation) and asexuality (when only phylogenetically clustered asexuals were considered), but did not propose any mechanistic explanation for these findings.

Considering the many species and supra‐specific taxa in soil that reproduce via parthenogenesis, we assume that identifying factors favoring parthenogenesis in these species may help in understanding why sexual reproduction predominates in the animal kingdom. With the overarching goal of testing the predictions of the RQT and SRTS, we specifically aimed at identifying (a) habitats in which parthenogenetic/ sexual reproduction dominates, and (b) macro‐ecological patterns along latitudinal and altitudinal gradients, as well as community characteristics, such as density and diversity, associated with the dominance of parthenogenetic/ sexual reproduction. Based on the SRTS, making explicit predictions on environmental factors favoring sexual/ parthenogenetic reproduction, we expected parthenogenetic reproduction to predominate in ecosystems where resources are in ample supply and in disturbed habitats in which populations are likely to be structured predominantly by density‐independent factors.

## 2 MATERIAL AND METHODS

We collected data from 25 studies that had investigated oribatid mite communities in different ecosystems and habitats (Table S1). We included only studies where all or most oribatid mites were identified to species level, where total density of oribatid mites was given, and from which we could retrieve data on altitude and latitude. In most cases, we contacted the first author of the study and asked for the original data. In few cases, we estimated density from other studies investigating similar habitats. We further included data from unpublished studies (Ricarda Lehmitz, unpubl. data; Mark Maraun, unpubl. data). The selected studies represent the range of environments in which oribatid mites occur and cover various biomes including temperate and tropical forests, meadows, fields, freshwater, salt marshes, and peat bogs, and microhabitats such as deadwood, mesovoid shallow substratum (a system of empty spaces within the stony debris covered by soil), and littoral algal mats.

The reproductive mode of individual species was either taken from literature (Cianciolo & Norton, 2006; Domes, Scheu, & Maraun, 2007; Domes‐Wehner, 2009; Fischer, Schatz, & Maraun, 2010; Grandjean, 1965; Norton et al., 1993; Palmer & Norton, 1991; Wehner, Scheu, & Maraun, 2014) or inferred from the reproductive mode of closely related species (see Table S1, for details).

To visualize patterns of community dissimilarity and identify factors correlating with species composition across different biomes and habitats, we used detrended correspondence analysis (DCA) as implemented in CANOCO 5 (Microcomputer Power, Ithaca, USA, 2012). Altitude, latitude, the percentage of parthenogenetic oribatid mite individuals, the percentage of parthenogenetic oribatid mite species, oribatid mite species number, and oribatid mite densities were included as passive/supplementary variables.

Further, we used linear regression to relate altitude, latitude, species richness, and total density of oribatid mites to the percentage of parthenogenetic individuals and parthenogenetic species. In these regression analyses, each community represented a single data point. To improve normality and homogeneity of variance, data were log‐transformed. Residuals were tested for heteroscedasticity and normality. As the relationships analyzed, at least in part, might be due to phylogenetic relatedness of species, we controlled for phylogenetic relatedness in the analysis of the data. Unfortunately, however, a solid phylogeny of the very wide range of taxa included in this study does not exist precluding the use of standard phylogenetic comparative methods (Felsenstein, 1985; Martins & Hansen, 1997; Swenson, 2014). Confronted with this limitation, we used the taxonomic system of oribatid mites as surrogate of the phylogeny. We assume this to be justified as molecular phylogenies of oribatid mites in general match the established taxonomic system (Pachl et al., 2017; Schaefer et al., 2010). We assigned each species to genus, family, superfamily, and suborder, and assumed that species within the same genus where phylogenetically closer to each other than species of different genera, and assumed that this is also true for higher taxonomic ranks. We then assigned an arbitrary unitary distance to species within the same genus and accordingly created an ultrametric tree based on taxonomic distance. We converted the tree into a cophenetic distance matrix and calculated mean taxonomic distance between each pair of communities (equivalent to phylogenetic beta diversity). We then subjected the resulting community distance matrix to Principal Coordinate Analysis and extracted as many axes as needed to account for at least 2/3 of the variance in the distance matrix. The axes were used as proxies for phylogenetic correlation between the analyzed communities and were thus used to approximate a phylogenetic correction of the variables analyzed with regression. The analyses were implemented using the R packages *ape*, *phylobase*, *picante*, *phytools,* and *Geiger* (R Development Core Team 2018).

## 3 RESULTS

**Figure 1:**
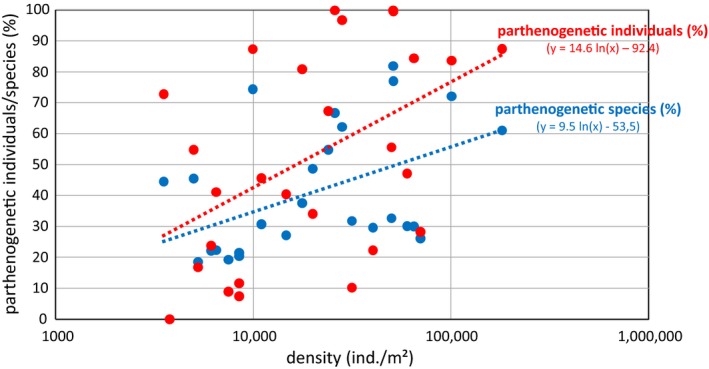
Relationship between oribatid mite density and the proportion of parthenogenetic individuals and species, respectively

Generally, the percentage of both parthenogenetic individuals and parthenogenetic species correlated significantly with the total density of oribatid mites (*F*_1,25_ = 4.97, *p* < 0.045 and *F*_1,25_ = 4.47, *p* < 0.044, respectively). On a logarithmic scale, a similar proportion of the variance in the number of individuals of parthenogenetic species (*r* ^2^ = 0.22) and the number of parthenogenetic species (*r* ^2^ = 0.23) was explained by density (Figure 1). Density was highest in peat bogs, acidic forests and in freshwater systems, and these were the ecosystems in which parthenogenetic species dominate (Table S1). The correlation between total density and frequency of parthenogenesis also held when corrected for phylogenetic relatedness as approximated by the first six axes of PCoA of the taxonomic distance matrix (Figure S1 and Table S2). Of these six axes, only axis 2 significantly correlated with density and percentage of parthenogenetic species and individuals (Table S2). We thus regressed these three variables against PCoA axis 2 and used the residuals as phylogenetically corrected variables (Figure S1). As expected, the *r* ^2^‐values slightly decreased, but the slope of the regression lines remained statistically significant (*p* < 0.01).

In contrast to density, the reproductive mode of oribatid mites did not significantly correlate with species richness (total number of species present) (*r* ^2^ = 0.09, *F*_1,25_ = 2.68, *p* = 0.114 and *r* ^2^ = 0.10, *F*_1,25_ = 2.98, *p* = 0.096 for the number of individuals of parthenogenetic species and the number of parthenogenetic species, respectively). Communities in tropical lowland as well as montane ecosystems (Indonesia, Ecuador 1,000 m, Vietnam 1,500 m) were among the richest in number of species and were dominated by sexual species (Table S1). However, salt marshes in Europe with only few species also were predominantly colonized by sexual species.

Reproductive mode was not significantly correlated with latitude (*r* ^2^ = 0.08, *F*_1,25_ = 2.20, *p* = 0.15; *r* ^2^ = 0.05 and *F*_1,25_ = 1.22, *p* = 0.27 for the number of individuals of parthenogenetic species and the number of parthenogenetic species, respectively). At high latitudes, there were habitats with both high (peat bogs, freshwater) and low frequency of parthenogenetic species (dead wood, fungal sporocarps, canopy of trees; Table S1).

Reproductive mode also did not correlate significantly with altitude (*r* ^2^ = 0.004, *F*_1,25_ = 0.12, *p* = 0.72; *r* ^2^ < 0.001 and *F*_1,25_ = 0.014, *p* = 0.91 for the number of individuals of parthenogenetic species and the number of parthenogenetic species, respectively). At low and very high altitude (> 4,000 m) parthenogenetic species predominate, whereas at intermediate altitude (~3,000 m) there were many sexual taxa (Table S1) indicating a hump‐shaped relationship. However, this pattern was not consistent because parthenogenetic species dominate in temperate and boreal lowland regions, whereas at low altitudes in the tropics sexual species predominate.

**Figure 2:**
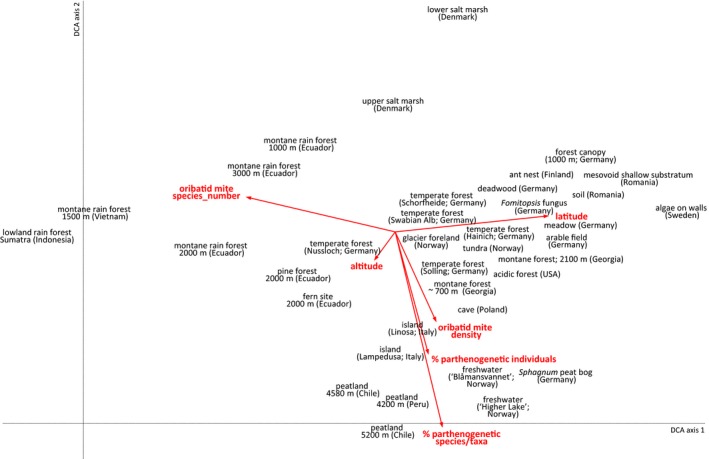
Detrended correspondence analysis (DCA) of oribatid mite species from 37 sites. Species number, altitude, latitude, density, percentage of parthenogenetic individuals, and percentage of parthenogenetic species were included as “passive/supplementary variables.” For clarity, oribatid mite species names were eliminated from the figure. For details of the habitats see Table S1. (Eigenvalues of axis 1 = 0.95 and axis 2 = 0.82, length of gradient 13.3; permutation test of first axis: *F* = 1.6; *p* = 0.002)

In the following, ecosystems and habitats are discussed and grouped according to the general pattern of reproductive mode of the oribatid mite communities (Figure 2). Ecosystems and habitats dominated by parthenogenetic species (50%–100% of all species) are discussed first, then the ones with intermediate numbers (25%–50%) and with few parthenogenetic species (<25%).

### 3.1 Ecosystems and habitats dominated by parthenogenetic species

Both the number of parthenogenetic oribatid mite species as well as the number of individuals of parthenogenetic species dominated in freshwater systems, peat bogs, acidic forests and in glacier foreland (Table S1). In freshwater systems, the Brachypylina genera *Hydrozetes* and *Limnozetes* dominated. All known species of *Limnozetes* are parthenogenetic while *Hydrozetes* includes both parthenogenetic and sexual species. Other common species in these habitats (e.g., *Tyrphonothrus maior*, *Trhypochthoniellus longisetosus*, *Platynothrus peltifer*) belong to large parthenogenetic taxa within Nothrina (formerly Desmonomata). Parthenogenetic species also dominate in peat bogs and acidic forests (temperate and boreal); members of Nothrina, Enarthronota, Suctobelbidae, *Oppiella nova,* and *Tectocepheus* spp. are the main taxa. In young postglacier foreland (about 40 years after retreat of glacier), oribatid mites are also dominated by parthenogenetic species such as *Tectocepheus velatus*, *Oppiella nova* and Suctobelbidae, Enarthronota and Nothrina. Total oribatid mite density in all these ecosystems was high, typically > 50,000 ind./m^2^.

### 3.2 Ecosystems and habitats with intermediate numbers of parthenogenetic species

Systems with similar numbers of parthenogenetic and sexual taxa (and individuals) included moss‐grass tundra, temperate base‐rich forests, meadows, fields, tropical mountain rain forests, salt marshes, and algal mats (Table S1). In moss–grass tundra (but not in lichen‐dominated tundra), parthenogenetic species of the taxa Enarthronota, Nothrina, and *Tectocepheus* make up about 40% of all species. In base‐rich temperate forests (with high macrofauna activity), in temperate grasslands, meadows and at agricultural sites, the proportion of parthenogenetic species is intermediate. Overall, compared to acidic forests, peat bogs, and tundra, the proportion of the early‐ (Enarthronota) and middle‐derivative (Nothrina) oribatid mites is much lower, and many species in these systems are from the derived Brachypylina. Parthenogenetic species predominantly comprise the Brachypylina taxa Oppiidae and Suctobelbidae. In tropical rain forests (e.g., lowland Indonesia, Ecuador 1,000 m, Vietnam 1,500 m), about 40% of all species are from parthenogenetic species including the generalistic *O. nova* and Suctobelbidae, but also other Brachypylina species, for example, species of the genera *Rostrozetes*, *Protoribates*, *Berlesezetes,* and *Licnozete*s.

### 3.3 Ecosystems and habitats with low numbers of parthenogenetic species

Both the number of sexual oribatid mite species as well as the number of individuals of sexual species dominated in deadwood, fungal sporocarps, tree canopies, tropical forests, temperate montane rain forests, subterranean sites (caves, mesovoid shallow substratum, deep soil), and on islands (Table S1). Oribatid mite species colonizing lichens, mosses, fungi (which often colonize substrates such as dead wood), and other living resources are predominantly sexual. Sexual species/taxa such as *Phauloppia* spp., *Jugatala* spp., *Mycobates* spp., *Carabodes labyrinthicus*, *C. willmanni*, *Cymberemaeus cymba,* and *Micreremus brevipes* dominated in lichens (as indicated by stable isotopes or observational studies). Oribatid mites in sporocarps of fungi also are mainly sexual species from the genera *Carabodes*, *Siculobata*, *Caleremaeus*, *Autogneta* and from the family Neoliodidae. Similarly, oribatid mites associated with mosses include mainly sexual species of the genera *Minunthozetes*, *Melanozetes*, *Edwardzetes*, *Maudheimia,* and *Ameronothrus*. Sexual oribatid mites predominate on the bark of trees, mainly comprising species feeding on lichens, algae, mosses, and fungi.

Generally, the fraction of individuals of sexual oribatid mite species (as percentages of total) in caves is high. Typical cave dwellers, for example, *Gemmazete*s, *Hypogeoppia* are all sexual. Similarly, in mesovoid shallow substratum sexual species (95%) and individuals (99%) dominate. Species occurring frequently in mesovoid shallow substratum were *Ceratoppia bipilis*, *Oribatella longispina*, *Chamobates birulai,* and *Pilogalumna tenuiclava* (Nae & Bancila, 2017). Oribatid mite densities in all of the above‐mentioned habitats are low.

## 4 DISCUSSION

The proportion of parthenogenetic species and individuals correlated positively with oribatid mite density being high in peat bogs at high and low altitude, in freshwater systems and in acidic forests (Huhta et al., 1986; Maraun & Scheu, 2000; Murvanidze & Kvavadze, 2009; Murvanidze, Mumladze, Arabuli, & Kvavadze, 2011). Other factors such as latitude, altitude, or species number did not correlate with the frequency of parthenogenetic species or individuals in oribatid mite communities. This supports the view that parthenogenetic species flourish in habitats where resources are plentiful and/or easily accessible supporting large populations, and this is consistent with predictions of the SRTS. Generally, the observed patterns suggest that the mode of reproduction is related to bottom‐up rather than top‐down regulation of populations, which argues against the RQT but supports the SRTS (Maraun, Norton, Ehnes, Scheu, & Erdmann, 2012; Scheu & Drossel, 2007).

Species numbers of oribatid mites correlated poorly with the reproductive mode of oribatid mites and this is consistent with earlier findings. Both species‐rich (e.g., tropical lowland sites in Indonesia) and species‐poor communities (e.g., salt marsh habitats; Winter, Haynert, Scheu, & Maraun, 2018) may be dominated by sexual species. Similarly, both species‐poor (e.g., high Andeans region) and species‐rich communities (e.g., acidic forests) may be dominated by parthenogenetic species. The fact that species‐rich communities, where biotic interactions are likely to be more frequent and more pronounced than in species‐poor communities, at least in part are dominated by parthenogenetic species argues against the RQT.

Altitude did not correlate with reproductive mode in oribatid mites. This was due to the fact that low‐altitude forests in the tropics harbor few parthenogenetic species whereas acidic temperate forests harbor many (Table S1). Furthermore, the percentage of sexual species (as well as individuals) decreases with increasing altitude up to about 3,000 m in tropical as well as temperate regions (Fischer, Meyer, & Maraun, 2014; Maraun et al., 2013), but in the Andes at 4,000–5000 m it is lower than at 3,000 m (Covarrubias, 2009; Hense, 2016; Maraun et al., 2013). The dominance of parthenogenetic species at very high altitudes in the Andean mountains probably is due to the fact that peatland vegetation dominates at these sites which are colonized by oribatid mite communities similar to those of peat bogs from lowland sites at high latitudes (Lehmitz & Maraun, 2016; Mumladze, Murvanidze, & Behan‐Pelletier, 2013; Seniczak, Seniczak, Maraun, Graczyk, & Mistrzak, 2016). The reduced frequency of sexual species at very high altitude conforms to predictions of both the RQT as well as the SRTS since they suggest that harsh environmental conditions foster parthenogenesis by reducing parasite–host interactions and by reducing density‐dependent population regulation, respectively. From the perspective of the RQT, the increase in the dominance of sexual species up to 3,000 m argues for increased parasite–host/ predator–prey interactions up to this altitude which is unlikely, in particular considering that above 3,000 m the pattern is reversed. From the perspective of the SRTS, it argues for increased resource limitation and/or more simplified habitat structure at higher altitude which appears to be more plausible. In fact, the density of oribatid mites declines in both temperate and tropical regions up to about 3,000 m indicating increased resource shortage (Fischer et al., 2014; Maraun et al., 2013; Marian, Sandmann, Krashevska, Maraun, & Scheu, 2018).

Latitude also did not correlate with the proportion of parthenogenetic individuals or species. This was due to the fact that there are sites at low latitude with few (high Andes) and many sexual species (lowland tropical sites), and also sites at high latitude with few (peatland) or many (dead wood, forest canopy) sexual species. Presumably, this is due to the fact that abiotic conditions at low latitudes (e.g., peat bogs in Germany and Poland) and high altitudes (e.g., high Andes) resemble each other, arguing that habitat‐specific factors, such as those, for example, in peatlands, rather than latitude drives the reproductive mode of oribatid mites. From the perspective of the RQT and SRTS, the lack of correlation between latitude and reproductive mode in oribatid mites suggests that neither parasite–host/ predator–prey interactions nor resource shortage/ habitat heterogeneity changes in a uniform way with latitude.

Ecosystems dominated by parthenogenetic species and individuals of oribatid mites included freshwater, peat bogs, acidic forests, and glacier foreland. In these ecosystems, oribatid mite densities typically are high. As discussed above, these findings generally support the SRTS. Overall, the fact that ecosystems where food resources (i.e., dead organic material) accumulate (peat bogs, acidic forests) or are made freshly available (glacier foreland) or are generally plentiful (periphyton in freshwater) are dominated by parthenogenetic individuals/species supports the STRS.

Ecosystems with intermediate numbers of parthenogenetic individuals/species (e.g., moss‐grass tundra, temperate base‐rich forests meadows, fields, tropical mountain rain forests, algal mats) are more difficult to interpret in respect to predictions of the RQT and SRTS. However, in some of the systems (e.g., tundra, algal mats) abiotic conditions are harsh and therefore populations are likely structured by density‐independent factors (e.g., freezing) which supports the view that parthenogenetic individuals/species flourish if resources are not fully exploited due to high death rates (Scheu & Drossel, 2007).

Oribatid mite communities are dominated by sexual species in deadwood, fungi, deep soil, canopy, islands, tropical forests, temperate mountain forests, caves, mesovoid shallow substratum, and in deep soil. In a number of these habitats, oribatid mite species predominate which feed on living food resources such as mosses, fungi, lichens, and algae (Caruso, Noto La Diega, & Bernini, 2005), and this presumably also is true for those living on deadwood or in the canopy of trees (Wehner, Heethoff, & Brückner, 2018). In fact, stable isotope (^15^N, ^13^C) studies suggest that oribatid mites on the bark of trees as well as in fungal sporocarps feed on lichens, mosses, algae, and fungi (Bluhm, Scheu, & Maraun, 2015; Fischer et al., 2010; Maraun, Augustin, Müller, Bässler, & Scheu, 2014), and these species typically reproduce sexually. As feeding on living food resources may be associated with increased bottom‐up population regulation, for example, due to defense mechanisms of the consumed species, the predominance of sexual reproduction is consistent with assumptions of the SRTS, whereas feeding on living resources is unlikely to be related to stronger top‐down processes and therefore unlikely to support the RQT.

Most species and individuals of oribatid mites in caves are sexual (Ducarme, Wauthy, André, & Lebrun, 2004; Ivan & Vasiliu, 2010); however, close to the cave entrance or in flooded caves there are more parthenogenetic species (Maslak & Barczyk, 2011). Bruckner (1995) found many Damaeidae and Oppiidae (overall 49 species) in caves, most of which reproduce sexually. The few collected parthenogenetic species, for example, *Platynothrus peltifer*, usually occur close to the entrance of the caves. Ducarme et al. (2004) found 33 species of oribatid mites in caves in Belgium, with mostly sexual Oppiidae dominating. Murvanidze (2014) collected 67 oribatid mite species in Georgian caves of which only four were parthenogenetic. Densities of oribatid mites in caves and in mesovoid shallow substratum are generally low (Ducarme et al., 2004; Ioana Nae, unpubl. data). The same applies to deep soil where densities are low and sexuals dominate (Ducarme et al., 2004). Again, these patterns argue for resource‐based processes rather than parasite–host/ predator–prey processes favoring sexual reproduction as low density suggests low resource supply, and the limited access of prey species in caves and deep in soil suggests top‐down control to be of limited importance.

## 5 CONCLUSIONS

In sum, (a) parthenogenetic oribatid mites dominate in habitats where densities are high, that is, where resources are plentiful and/or are easy to access, such as peat bogs and acidic forests. These habitats are characterized by the accumulation of organic matter and may resemble conditions in the Carboniferous when resources for decomposers were plentiful, coinciding with the main radiation of the predominantly parthenogenetic Nothrina (Schaefer et al., 2010). Supporting the dominant role of resources in controlling the reproductive mode of oribatid mites, other habitats rich in resources, such as glacier foreland, with plenty of unused resources, and freshwater habitats, rich in periphyton easily available for oribatid mites, also are characterized by many parthenogenetic species and individuals. (b) The number of individuals of parthenogenetic and sexual species is in a similar range in tundra, tropical lowland, and base‐rich temperate forests as well as in grassland and agricultural sites, where resources are less abundant and where population dynamics are likely to be driven by harsh abiotic conditions or human disturbance. (c) Sexual taxa dominate in systems where resources of oribatid mites are of low quality such as tropical montane rain forests, and where resource accessibility is limited by, for example, flooding (salt marsh) and drying (ephemeral freshwater ponds), as well as in systems where oribatid mites feed on living resources (lichens, fungi, mosses) that may defend themselves or where resources are generally limited (caves, deep soil). All these cases are consistent with the assumption of the SRTS that sexual reproduction is favored if food resources are in limited supply. Notably, the predominance of sexual reproduction in habitats where oribatid mite densities are low is surprising from the perspective of mating/sperm transfer as this is more difficult at low density and therefore should favor parthenogenetic reproduction, but argues that even when rare sexual species are not sperm limited.

## CONFLICT OF INTEREST

None declared.

## AUTHOR CONTRIBUTIONS

All authors contributed to the conception of the study, the acquisition of data, and the data analysis; they all drafted and revised the article critically and approved the final version to be published.

## Data Availability

Data are available from the Dryad Digital Repository (https://doi.org/10.5061/dryad.gr6qb4h).
