# Corrigendum

**DOI:** 10.1002/ece3.7078

**Published:** 2021-03-01

In “Parthenogenetic *vs*. sexual reproduction in oribatid mite communities” (Maraun et al., 2019), the authors have discovered errors in the text and the units of some of the data in their published article. The corrected text and Figures 1 and 2 are outlined below. The article has also been updated online.

**Figure 1:**
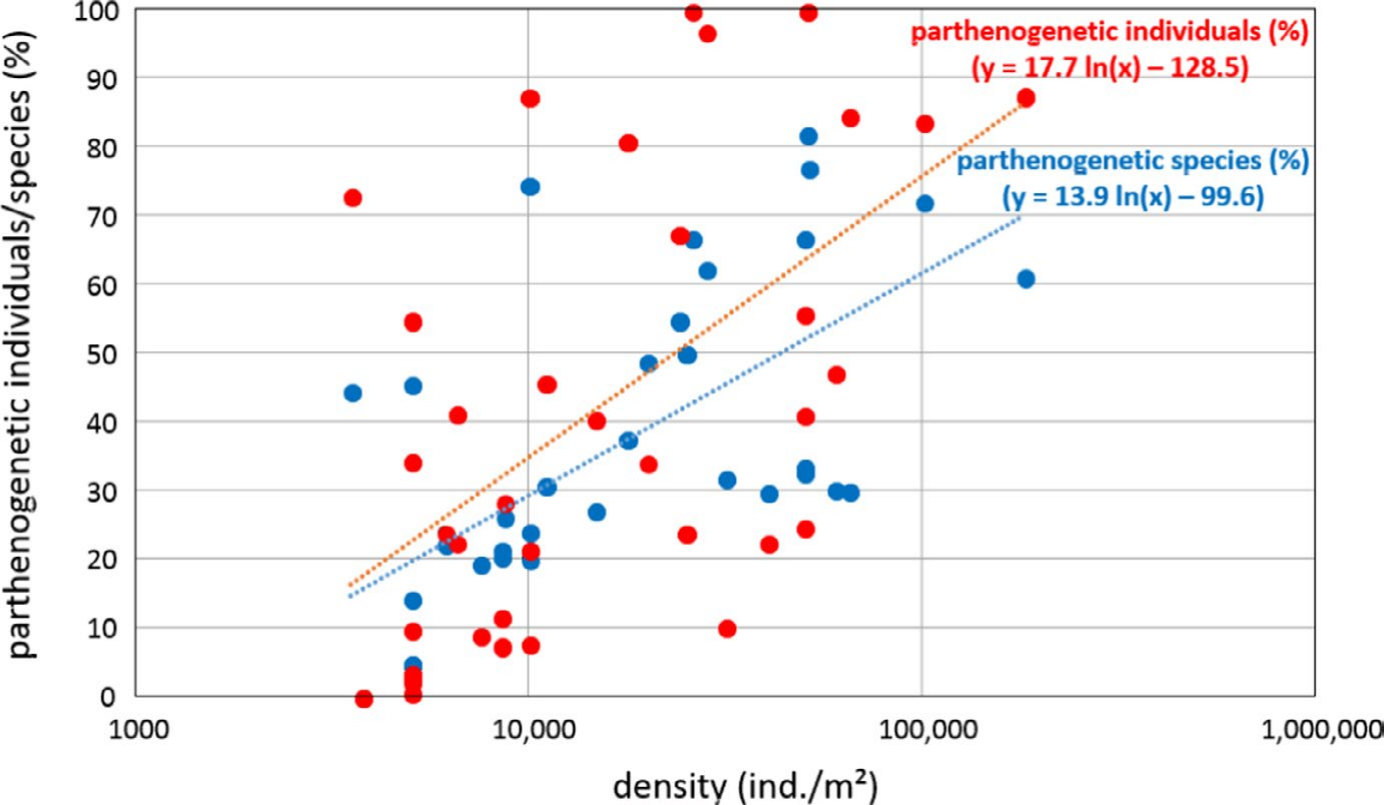
Relationship between oribatid mite density and the proportion of parthenogenetic individuals and species, respectively

**Figure 2:**
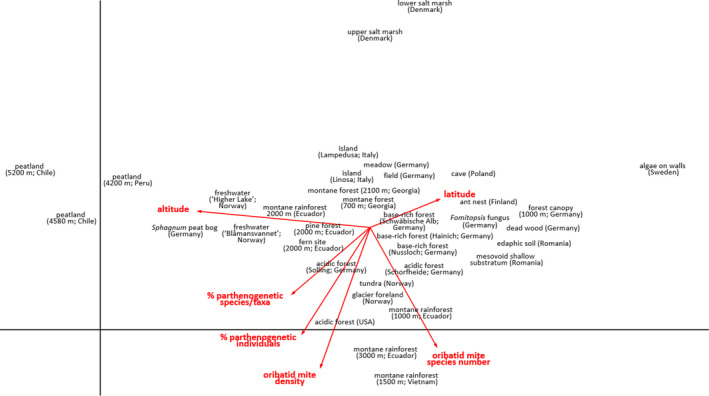
Detrended correspondence analysis (DCA) of oribatid mite species from 36 sites. Species number, altitude, latitude, density, percentage of parthenogenetic individuals, and percentage of parthenogenetic species were included as “passive/supplementary variables.” For clarity, oribatid mite species names were eliminated from the figure. For details of the habitats, see Table S1. (Eigenvalues of axis 1 = .98 and axis 2 = .94, length of gradient 19.7 and 15.4, respectively; permutation test of first axis: *F* = 1.4; *p* = .002)

**Abstract section:**

Overall, the data showed that low density of oribatid mites due to harsh environmental conditions is associated with high frequency of parthenogenesis supporting predictions of the Structured Resource Theory of Sex rather than the Red Queen hypothesis.

Should read as:

Overall, the data showed that high density of oribatid mites, indicating abundance of resources, is associated with high frequency of parthenogenesis supporting predictions of the Structured Resource Theory of Sex rather than the Red Queen hypothesis.

**In section 3 Results:**

1st paragraph:

“(*F*_1,25_ = 4.97, *p* < .045 and *F*_1,25_ = 4.47, *p* < .044, respectively).”

Should be replaced with:

“(*F*_1,34_ = 10.57, *p* < 0.0026 and *F*_1,34_ = 11.12, *p* < 0.0021, respectively).”

“(*r* ^2^ = .22)” and “(*r* ^2^ = .23)” should be “(*r* ^2^ = 0.41)” and “(*r* ^2^ = 0.31)”

2nd paragraph:

“(*r* ^2^ = .09, *F*_1,25_ = 2.68, *p* = .114 and *r* ^2^ = .10, *F*_1,25_ = 2.98, *p* = .096)”

Should be replaced by:

“(*r* ^2^ = 0.04, *F*_1,34_ = 1.49, *p* = 0.23 and *r* ^2^ = 0.09, *F*_1,34_ = 3.53, *p* = 0.069)”

The text “Communities in tropical lowland as well as montane ecosystems (Indonesia, Ecuador 1,000 m, Vietnam 1,500 m)”

Should be changed to:

“Communities in tropical montane ecosystems (Ecuador 1,000 m, Vietnam 1,500 m)”

3rd paragraph:

“*r* ^2^ = .08, *F*_1,25_ = 2.20, *p* = .15; *r* ^2^ = .05 and *F*_1,25_ = 1.22, *p* = .27”

Should be replaced by:

“*r* ^2^ = 0.02, *F*_1,34_ = 0.65, *p* = 0.42 and *r* ^2^ = 0.0006, *F*_1,34_ = 0.02, *p* = 0.88”

4th paragraph:

“*r* ^2^ = .004, *F*_1,25_ = 0.12, *p* = .72; *r* ^2^ < .001 and *F*_1,25_ = 0.014, *p* = .91”

Should be replaced with:

“*r* ^2^ = 0.02, *F*_1,34_ = 0.64, *p* = 0.43 and *r* ^2^ < 0.001, *F*_1,34_ < 0.001, *p* = .99”

**In section 3.1, the last sentence:**

“In tropical rain forests (e.g., lowland Indonesia, Ecuador 1,000 m, Vietnam…”

Should read:

“In tropical rain forests (e.g., Ecuador 1,000 m, Vietnam…”

**In section 4, Discussion:**

In the second sentence of the second paragraph:

“Both species‐rich (e.g., tropical lowland sites in Indonesia)…”

Should read:

“Both species‐rich (e.g., tropical forests in Ecuador)…”

In the fourth paragraph, second sentence:

“lowland tropical sites” should read “tropical sites.”

**In section 5, Conclusions:**

“(b) The number of individuals of parthenogenetic and sexual species is in a similar range in tundra, tropical lowland, and base‐rich temperate forests as well as in grassland and agricultural sites, where resources are less abundant and where population dynamics are likely to be driven by harsh abiotic conditions or human disturbance.”

Should read as follows:

“(b) The number of individuals of parthenogenetic and sexual species is in a similar range in tundra, tropical regions, and base‐rich temperate forests as well as in grassland and agricultural sites, where resources are less abundant and where population dynamics are likely to be driven by harsh abiotic conditions or human disturbance.”

Corrected Figures 1 and 2 with their captions are below:
