# Supplementary material for: Parthenogenetic vs. sexual reproduction in oribatid mite communities
Source: Ecol Evol. 2019 May 29;9(12):7324–32. doi: 10.1002/ece3.5303 (PMC6662391; doi:10.1002/ece3.5303)
Supplement: Supplementary file 1 [file ECE3-9-7324-s001.pptx]

## Slide 1
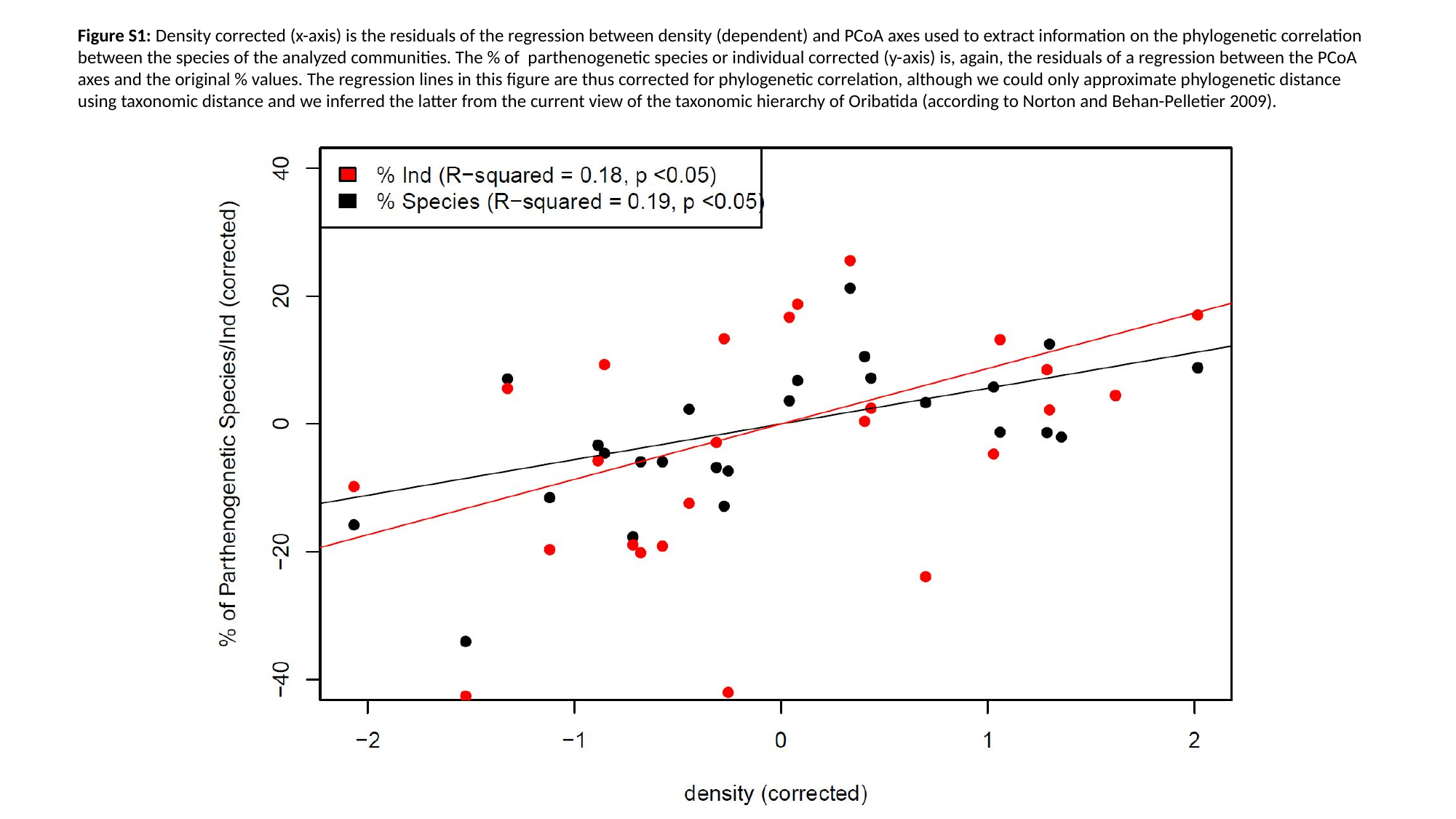

Figure S1: Density corrected (x-axis) is the residuals of the regression between density (dependent) and PCoA axes used to extract information on the phylogenetic correlation between the species of the analyzed communities. The % of parthenogenetic species or individual corrected (y-axis) is, again, the residuals of a regression between the PCoA axes and the original % values. The regression lines in this figure are thus corrected for phylogenetic correlation, although we could only approximate phylogenetic distance using taxonomic distance and we inferred the latter from the current view of the taxonomic hierarchy of Oribatida (according to Norton and Behan-Pelletier 2009).
