# Supplementary material for: Parthenogenetic vs. sexual reproduction in oribatid mite communities
Source: Ecol Evol. 2019 May 29;9(12):7324–32. doi: 10.1002/ece3.5303 (PMC6662391; doi:10.1002/ece3.5303)
Supplement: Supplementary file 3 [file ECE3-9-7324-s003.docx]

Table S2. Correlation matrix (Pearson’s coefficient) linking key variables (i.e., % of parthenogenetic individual and species, species number and total oribatid density) to each other and the first 6 PCoA axes extracted from the taxonomic distance matrix used to approximate phylogenetic relatedness of species. Bold values were statistically significant at p < 0.05. Given this matrix, we chose PCoA2 as the phylogenetic correction factors to regress % of parthenogenetic individual and % of parthenogenetic species against density (Fig. S1), species number and also altitude and latitude (not shown, correlations not significant).

|  | % indiv. | | % species | Species N | Density | PCoA1 | PCoA2 | PCoA3 | PCoA4 | PCoA5 | PCoA6 |
| --- | --- | --- | --- | --- | --- | --- | --- | --- | --- | --- | --- |
| % indiv. | | 1.00 | **0.86** | **-0.31** | **0.41** | -0.11 | **0.77** | -0.15 | -0.02 | 0.07 | -0.20 |
| % species | | **0.86** | 1.00 | -0.33 | **0.39** | 0.15 | **0.78** | -0.23 | 0.02 | 0.22 | -0.22 |
| Species N | | **-0.31** | **-0.33** | 1.00 | 0.06 | 0.16 | **-0.45** | -0.16 | -0.64 | 0.20 | 0.56 |
| Density | | **0.41** | **0.39** | 0.06 | 1.00 | 0.22 | **0.25** | -0.02 | 0.01 | 0.31 | -0.15 |
